# Transcriptomic and Proteomic Profiling of Human Stable and Unstable Carotid Atherosclerotic Plaques

**DOI:** 10.3389/fgene.2021.755507

**Published:** 2021-11-04

**Authors:** Mei-hua Bao, Ruo-qi Zhang, Xiao-shan Huang, Ji Zhou, Zhen Guo, Bao-feng Xu, Rui Liu

**Affiliations:** ^1^ Academician Workstation, Changsha, China; ^2^ School of Stomatology, Changsha Medical University, Changsha, China; ^3^ Department of Pharmacology, Changsha Health Vocational College, Changsha, China; ^4^ First Hospital of Jilin University, Changchun, Jilin, China; ^5^ Department of VIP Unit, China-Japan Union Hospital of Jilin University, Changchun, China

**Keywords:** atherosclerosis, unstable plaques, RNA-seq, proteome, transcriptome

## Abstract

Atherosclerosis is a chronic inflammatory disease with high prevalence and mortality. The rupture of atherosclerotic plaque is the main reason for the clinical events caused by atherosclerosis. Making clear the transcriptomic and proteomic profiles between the stabe and unstable atherosclerotic plaques is crucial to prevent the clinical manifestations. In the present study, 5 stable and 5 unstable human carotid atherosclerotic plaques were obtained by carotid endarterectomy. The samples were used for the whole transcriptome sequencing (RNA-Seq) by the Next-Generation Sequencing using the Illumina HiSeq, and for proteome analysis by HPLC-MS/MS. The lncRNA-targeted genes and circRNA-originated genes were identified by analyzing their location and sequence. Gene Ontology and KEGG enrichment was carried out to analyze the functions of differentially expressed RNAs and proteins. The protein-protein interactions (PPI) network was constructed by the online tool STRING. The consistency of transcriptome and proteome were analyzed, and the lncRNA/circRNA-miRNA-mRNA interactions were predicted. As a result, 202 mRNAs, 488 lncRNAs, 91 circRNAs, and 293 proteins were identified to be differentially expressed between stable and unstable atherosclerotic plaques. The 488 lncRNAs might target 381 protein-coding genes by *cis*-acting mechanisms. Sequence analysis indicated the 91 differentially expressed circRNAs were originated from 97 protein-coding genes. These differentially expressed RNAs and proteins were mainly enriched in the terms of the cellular response to stress or stimulus, the regulation of gene transcription, the immune response, the nervous system functions, the hematologic activities, and the endocrine system. These results were consistent with the previous reported data in the dataset GSE41571. Further analysis identified CD5L, S100A12, CKB (target gene of lncRNA MSTRG.11455.17), CEMIP (target gene of lncRNA MSTRG.12845), and SH3GLB1 (originated gene of hsacirc_000411) to be critical genes in regulating the stability of atherosclerotic plaques. Our results provided a comprehensive transcriptomic and proteomic knowledge on the stability of atherosclerotic plaques.

## Introduction

Atherosclerosis is a chronic inflammatory disease with high prevalence and mortality. The stability of atherosclerotic plaques is the main reason for its clinical manifestations. Unstable plaques, also known as vulnerable plaques, are characterized by a large lipid core, a thin fibrotic cap, less smooth muscle cells, less collagen, and elevated inflammation. The broken of unstable plaques blocks capillaries, forms thrombosis, and eventually triggers clinical manifestations, such as ischemic stroke and myocardial infarction. Figuring out the genes and proteins which play critical roles in the stability of atherosclerotic plaques is important.

Recent researches have demonstrated the functions of lncRNAs and circRNAs in atherosclerosis (Xiao et al., 2018; Fasolo et al., 2019; L.; Wang et al., 2019; Cao et al., 2020). For example, lncRNA FENDRR, LincRNA-p21, ANRIL, MIAT, CDK2B-AS1, PELATON participated in the formation and stability of atherosclerotic plaques. They interfered with the phagocytosis, lipid uptake, and reactive oxygen species formation during atherogenesis (Çekin et al., 2018; Hung et al., 2020; Ou et al., 2020). CircRNA is a plentiful, stable, diversified, and conserved class of non-coding RNA molecules that circularized from head to tail with a covalent bond of 5–3 (Jeck et al., 2013). It is involved in a wide range of biological and pathological processes, such as carcinogenesis and cardiovascular diseases. CircRNAs act as miRNA sponges, decoys, or scaffolds of gene expression (Poller et al., 2018). Studies reported the participation of cirRNA00044073, circRNA-PTPRA, circRNA_0003204, and circHIPK3 in atherosclerosis through affecting the autophagy, proliferation and invasion, and tube formation (Shen et al., 2019; Wei et al., 2020; Zhang, 2020; Zhang et al., 2020).

Recently, some studies have investigated the transcriptome profiles in atherosclerosis. In a microarray analysis, 236 lncRNAs and 488 mRNAs were identified to be differentially expressed in human advanced atherosclerotic plaques compared to the normal arterial intimae (Bai et al., 2019). An RNA-Seq identified 1,259 annotated and 381 new RNAs in coronary artery disease (Pan et al., 2019). A weighted gene correlation network analysis identified several key genes in ruptured atherosclerotic plaques and other aging diseases (Yang et al., 2016; Xu et al., 2020; Yang et al., 2020). However, no comprehensive study and analysis was performed on the whole transcriptome and proteome in stable and unstable plaques.

In our present study, we obtained the stable and unstable plaques from patients conducting carotid endarterectomy (CEA), measured the transcription profiles and protein profiles by RNA-Seq and HPLC-MS/MS, identified the differentially expressed (DE) mRNAs, lncRNAs, circRNAs, and proteins, analyzed the functions of these differentially expressed genes. The present study provided a comprehensive knowledge of the gene and protein expression profiles responsible for the stability of atherosclerotic plaques, and identified several essential RNAs and proteins.

## Methods and Materials

### Patients and Samples

The atherosclerotic plaques were obtained from 10 patients undergoing CEA operation in the First Hospital of Jilin University (Changchun, Jilin, China) from July 2019 to November 2019. The plaques were fast-frozen in liquid nitrogen and stored at −80°C. The classification of unstable or stable plaques was carried out according to the criteria of the American Heart Association (AHA) (Hetterich H et al., 2016). Briefly, type I/II: near-normal wall thickness, no calcification; Type III: diffuse intimal thickening or small eccentric plaque, no calcification; Type IV/V: plaque with lipid or necrotic core surrounded by fibrous tissue with possible calcification; Type VI: complex plaque with possible surface defect, hemorrhage, or thrombus; Type VII: calcified plaque; Type VIII: fibrotic plaque without lipid core and with possible small calcification. Type I-II, III, VII, VIII were considered stable, while type IV-V, VI to be unstable plaques. Two independent investigators conducted the plaque classification.

The procedures were approved by the Ethics Committee of the First Hospital of Jilin University (No. 2019-272, Changchun, Jilin). Written informed consent was obtained from every participant. Eventually, 5 stable plaques and 5 unstable plaques were obtained for further analysis. The stable or unstable plaque from each patient was divided into two parts evenly. One part was used for whole transcriptome sequencing (RNA-Seq), while the other part was for LC-MS/MS detection.

### RNA Extraction, Library Preparation, and RNA-Sequencing

The Next-Generation Sequencing (NGS) analysis was performed in the Shanghai Personalbio Technology Co., Ltd. (Shanghai, China). Total RNA was isolated using the Trizol reagent (Invitrogen, Carlsbad, CA, United States). The qualities and quantities of the RNA were measured using NanoDrop spectrophotometer (Thermo Scientific, Waltham, Massachusetts, United States). The integrity of the total RNA was determined by Bioanalyzer 2,100 (Agilent Technologies, Santa Clare, CA, United States) and 1% agarose gel electrophoresis.

Sequencing libraries were generated according to the following steps: poly-T oligo-attached magnetic beads were used to purify mRNA from total RNA. The mRNA was fragmented by divalent cations under elevated temperature in an Illumina proprietary fragmentation buffer. Then, the first strand cDNA was synthesized using random oligonucleotides and SuperScript II, followed by the second strand cDNA synthesis using DNA Polymerase I and RNase H. After adenylation of the 3’ ends of the DNA fragments, Illumina PE adapter oligonucleotides were ligated to prepare for hybridization. The cDNA fragments with a length of 400–500 bp were selected. Then the library fragments were purified using the AMPure XP system (Beckman Coulter, Beverly, CA, United States). DNA fragments with ligated adaptor molecules on both ends were amplified using Illumina PCR Primer Cocktail in a 15 cycle PCR reaction. The products were purified (AMPure XP system) and quantified using the Agilent high sensitivity DNA assay on a Bioanalyzer 2,100 system (Agilent). The sequencing library was then sequenced on NovaSeq 6,000 platform (Illumina) by Shanghai Personal Biotechnology Co. Ltd.

Samples are sequenced on the NovaSeq 6,000 platform to get image files, which were then transformed to the original data in FASTQ format (Raw Data). These Raw Data were filtered to get high-quality sequence (Clean Data) by Cutadapt (v1.15) software, which removed low-quality Reads and connectors. The reference genome and gene annotation files were downloaded from the genome website. The filtered reads were mapped to the reference genome (Homo-sapiens.GRCh38. dna.primary_ assembly. fa) using HISAT2 v2.0.5.

### Identification of Differentially Expressed mRNA, lncRNA, and circRNA

The expression of mRNAs was identified by the HTSeq (0.9.1) statistics. The Read Count values on each gene were considered to be the original expression level, and then the FPKM method was used to standardize them. The expression difference of mRNA between stable and unstable plaque groups was analyzed by DESeq (1.30.0).

The lncRNAs were identified by the Stringtie software. Briefly, the transcripts with length >200 bp and exon number≥2 were identified first. Transcriptions with the class code of x/u/i (x stand for antisense lncRNA, u stand for intergenic lncRNA, i stand for intronic lncRNA) were identified secondly. LncRNAs with coverage >3 were identified as expressed lncRNAs. The newly identified lncRNA was nominated by the Stringtie automatically with a title of “MSTRG”.

The remaining unmapped reads were considered to be candidates of circRNAs. On the candidate transcripts, 20 bp at each end was used as 5′ Anchor or 3’ Anchor. The Anchors were then mapped to the reference sequence. If the sequences of the Anchors were reverse to the mapped sequence and the junction was consistent with the splicing pattern of AG-GT, it was determined to be a circRNA. The expression level of circRNAs was calculated by the method of Transcripts Per kilobase of exon model per Million mapped reads (TPM).

The DESeq was used to analyze the DE mRNAs, lncRNAs, circRNAs. RNAs with |log2FoldChange| > 1.0 and *p*-value < 0.05 were identified as differentially expressed.

The MeV 4.9.0 software was used to perform clustering and visualizing the expression pattern of 20 DE mRNAs, lncRNAs, and circRNAs.

### Verification of Differentially Expressed mRNA, lncRNA, and circRNA

The expression level of 4 DE mRNA, 4 DE lncRNA, 4 DE circRNA were verified by qPCR. The total RNAs from 5 stable and 5 unstable plaques were extracted by Trizol (Takara, Dalian, China). After concentration and quality evaluation, the total RNAs were reverse transcripted to cDNA by the PrimeScript RT reagent Kit with gDNA Eraser (perfect real time) kits (Takara, Dalian, China). The PCR reactions were conducted by Applied Biosystems Quantstudio 5 system with the following program: 95 °C 30 s, followed by 40 cycles of 95°C 5 s, 60°C 30 s. The primers were provided by Shanghai Sangon Biological Engineering Co. Ltd. (Shanghai, China). The primers used in the present study were as following: CD163: forward 5′-GGA TCT GCT GAC TTC AGA AG -3′, reverse 5′-CTC CTT GTC TGT TCC TCC AA-3’; (antisense); S100A8: Forward 5′-ATG CCG TCT ACA GGG ATG ACC T-3′, reverse 5′- AGA ATG AGG AAC TCC TGG AAG TTA-3’; FGF14: forward 5′-TAT TGC AGG CAA GGC TAC TAC TTG-3′, reverse 5′-GTT TTC ACT CCC TGG ATG GCA AC-3’; CDH19: forward 5′-ATT GGT CAG CCA GGA GCG TTG T-3′, reverse 5′- GCA GAT TCA GAG ACA GTC AAG CG-3’. lncRNA ENST00000430222 forward 5′- TCT CAA GTC GCT GAC ACC TCC TC-3′, reverse 5′- GGG TTG CCG AGT GAA GCT AAG AC-3'; lncRNA ENST0000062895 forward 5′-GCA AGG CGT CCG AAG TAT GAG TC-3′, reverse 5′-CGT CAG TAG AAG TTA GGC GAT CAG C -3'; lncRNA ENST00000631338 forward 5′-AGT TCA TCA CGG CTG CTG CTA AC-3′, reverse 5′- CTT GGC TTG GAG GGA GAA GAA TCA C-3'; lncRNA MSTRG18183 forward 5′-CCA GAG AGG AGG AAG AGG GGA ATC-3′, reverse 5′-TTA GGT GGG TGG AAG GCA GAG ATC-3'; hsacirc_013041 forward 5′-TGG TGT ATG CAA GTG GCC-3′, reverse 5′-TGC TGA AAA GCC AAC TGC TGG GTA G-3'; hsacirc_025902 forward 5′-AGA CCG TGG TGG TCA TCC-3′, reverse 5′-CCT GAG CCT TGA GAT AGT T-3'; hsacirc_054182 forward 5′-CAG AGC CAG CAT TCT TTC C-3′, reverse 5′-GAG CCT GTG GAT GAA GTG AG-3'; hsacirc_037511 forward 5′- CCC TAA AGA AAA TTG CTA-3′, reverse 5′- TTA TCA CAA ATC TCA GCC -3'. GAPDH forward: 5′-CTC TGC TCC TCC TGT TCG AC-3′, reverse: 5′-GCG CCC AAT ACG ACC AAA TC-3'. All samples were run in triplicate, and the results were analyzed using the 2^(−ΔΔCt)^ Method.

### The lncRNA-Targeted Genes and circRNA-Originated Genes Identification

As reported previously, the *cis*-acting regulation is an important mechanism of lncRNA. Through this, the lncRNAs activate, repress, or modulate the expression of neighboring target genes (Lam et al., 2014). These lncRNAs are treated as *cis*-acting lncRNAs. Usually, the *cis*-acting lncRNAs regulate the expression of their neighboring genes in a manner dependent on the location of their own sites of transcription. Therefore, the protein-coding genes neighboring the *cis*-acting lncRNAs might be their targets. Here, we searched the protein-coding genes within a distance of 1 kb ∼ −1 kb to the potential *cis*-acting lncRNAs. These protein-coding genes were considered to be the lncRNA-target genes of the corresponding lncRNA.

To predict the functions or mechanisms of circRNAs, we identified the circRNA-originated genes based on the theory that many circRNAs are originated from protein-coding genes and contain exonic sequences (Guo et al., 2014; You et al., 2015). Therefore, we analyzed the sequence of the differentially expressed circRNAs, identified the circRNAs with exons of protein-coding genes. These protein-coding genes were considered to be the originate genes of the corresponding circRNAs.

### Proteomic Analysis of Differentially Expressed Proteins in Stable and Unstable Plaques

Extraction and digestion of proteins: The samples were lysed by SDT solution (4% SDS, 100 mM Tris/HCl pH 7.6, 0.1 M DTT). The protein concentration was determined by BCA method. The extracted total proteins were hydrolyzed by trypsin using filter aided proteome preparation (FASP). The desalination was conducted on the C_18_ cartridge. After freeze-drying, the peptide was dissolved in 40 μl Dissolution buffer and quantified by OD280.

LC-MS/MS detection: Trypsin-digested peptides were separated using the Easy nLC nanoHPLC system. 2 μg of the sample were loaded with a constant flow of 4 μl/min on a Thermo Scientific EASY column C_18_ column. After trap enrichment, the peptides were eluted in the Easy C_18_ nanocolumn (75 μm*10 cm) by a linear gradient of solvent A (0.1% formic acid solution) and solvent B [0.1% formic acid in acetonitrile (84%)] with a constant flow of 250 nL/min. The solvent B changed from 0 to 35% in 0–50 min, from 35 to 100% in 50–58 min, and 100% in 58–60 min.

The HPLC system was coupled to an OrbiTrap QExactive mass spectrometer (Thermo Fisher Scientific Inc.) via an EasySpray source. The full scan MS survey spectra was m/z 300 to 1800 in positive mode. The first-grade mass spectrometry resolution was 70,000 at m/z 200. The automatic gain control (AGC) target was 3e6. The maximum IT was 10 ms. The dynamic exclusion was 40.0 s. The charge to mass ratio of peptides was collected under the following conditions: after a full scan, 10 MS2 scan was obtained; The MS2 activation type was HCD, with an isolation window of 2 m/z; The resolution of MS2 was 17,500 at 200 m/z; The normalized collision energy was 30 eV, and the underfill ratio was 0.1%.

Annotation and quantification of proteins: The raw file was annotated by Maxquant software (version 1.5.5.1). The parameters used for the analysis was as following: main search ppm was 6; max missed cleavages was 2; De-isotopic was True; enzyme was trypsin; fixed modifications was carbamidomethyl; variable modifications were oxidation, and the database was uniport_Homo_sapiens_186616_20191202; the decoy database pattern was Reverse; the label-free quantification (LFQ) was True; the peptide mass tolerance was ±20 ppm; the peptide FDR was≤0.01, and the protein FDR was≤0.01.

The differentially expressed proteins (DEPs) between stable and unstable plaques were identified by the criteria of |log2FoldChange| > 1.0 and *p*-value < 0.05.

### Gene Ontology and Kyoto Encyclopedia of Genes and Genomes pathway analysis of Differentially Expressed mRNAs, lncRNA-targeted genes, circRNA-originated genes, and Differentially Expressed Proteins.

GO enrichment analysis and KEGG pathway analysis were used to analyze the functions of DE mRNAs, lncRNA-targeted genes, circRNAs-originated genes, and DEPs. The DAVID online tool (https://david.ncifcrf.gov/) was used for GO and KEGG enrichment. The GO enriched genes into three annotations: biological process (BP), cell components (CC), and molecular function (MF). A *p*-value < 0.05 was considered to be significantly related GO terms or KEGG pathways.

### Protein-Protein Interaction Analysis of the Differentially Expressed Proteins

To identified the interactions between DEPs, the STRING online tool (website: https://string-db.org/) was used. The STRING database covers 9′643′763 proteins from 2′031 organisms. It provides direct (physical) interactions and indirect (functional) associations between proteins based on the computational prediction, knowledge transfer between organisms, and interactions aggregated from other (primary) databases. After the PPI analysis by STRING, the key clusters in the PPI network were analyzed by the tool of MCODE in Cytoscape software 3.8.3.

### Comparison Analysis of Transcriptome and Proteome, and Previously Reported Data Profile GSE41571.

A previous study reported a low correlation between transcripts and proteins (Ghazalpour et al., 2011). To analyze the consistency of expression pattern between transcriptome and proteome in the present study, and to identify the key genes which may play critical roles in the stability of atherosclerotic plaques, we analyzed the overlapped genes between DE mRNAs and DEPs. We also analyzed the relationship between lncRNA-targeted genes (identified in *The lncRNA-Targeted Genes and circRNA-Originated Genes Identification*) and the DEPs, the circRNA-originated genes (identified in *The lncRNA-Targeted Genes and circRNA-Originated Genes Identification*) and DEPs. The overlapped genes and the expression levels were summarized.

To verify our transcriptomic and proteomic results, we also downloaded the gene expression profile GSE41571 from the Gene Expression Omnibus (GEO, https://www.ncbi.nlm.nih.gov/geo/). The dataset GSE41571 contains 5 unstable and 6 stable atherosclerotic plaques obtained from CEA. These plaques were carried out genome-wide gene expression profiling using microarrays. The GEO2R tool on the GEO website was used to screen out the differentially expressed genes in GSE41571. Then we analyzed the overlapped genes between DE mRNAs from GSE41571 and our transcriptomic and proteomic profiles.

### LncRNA/circRNA-miRNA-mRNA Network Analysis

The lncRNAs and cirRNAs are involved in the gene regulation by many methods, such as binding to target genes, affecting the histone modification, activating transcriptional factors, and binding to miRNA as competitive endogenous RNA (ceRNA). To further analyze the functions of specific lncRNAs or circRNA, the miRNAs which interacted with the lncRNA/circRNA were analyzed by miRDB (http://mirdb.org/). Then the miRNA targeted mRNA was analyzed by miRDB. Cytoscape software 3.8.3 was used to visualize the lncRNA/circRNA-miRNA-mRNA network.

### Statistical Analysis

The data were presented in the form of mean ± S.D. *p*-value < 0.05 is considered statistically significant.

## Results

### Patients and Samples Information

A total of 5 patients with stable plaques and 5 patients with unstable plaques were involved in the present study. The characteristic information of these patients were shown in Supplementary Table S1. There was no significant difference between the two groups on age, sex, body weight index, smoke, alcohol, and lipid profiles. In the unstable plaque group, 40% of the patients are taking antihypertensives, antihyperlipidemic drugs, or antiplatelet drugs. In the stable plaque group, 40% of the patients are taking antihypertensives.

### The DE mRNAs, lncRNAs, circRNAs, and Proteins in Stable and Unstable Plaques.

**FIGURE 1:**
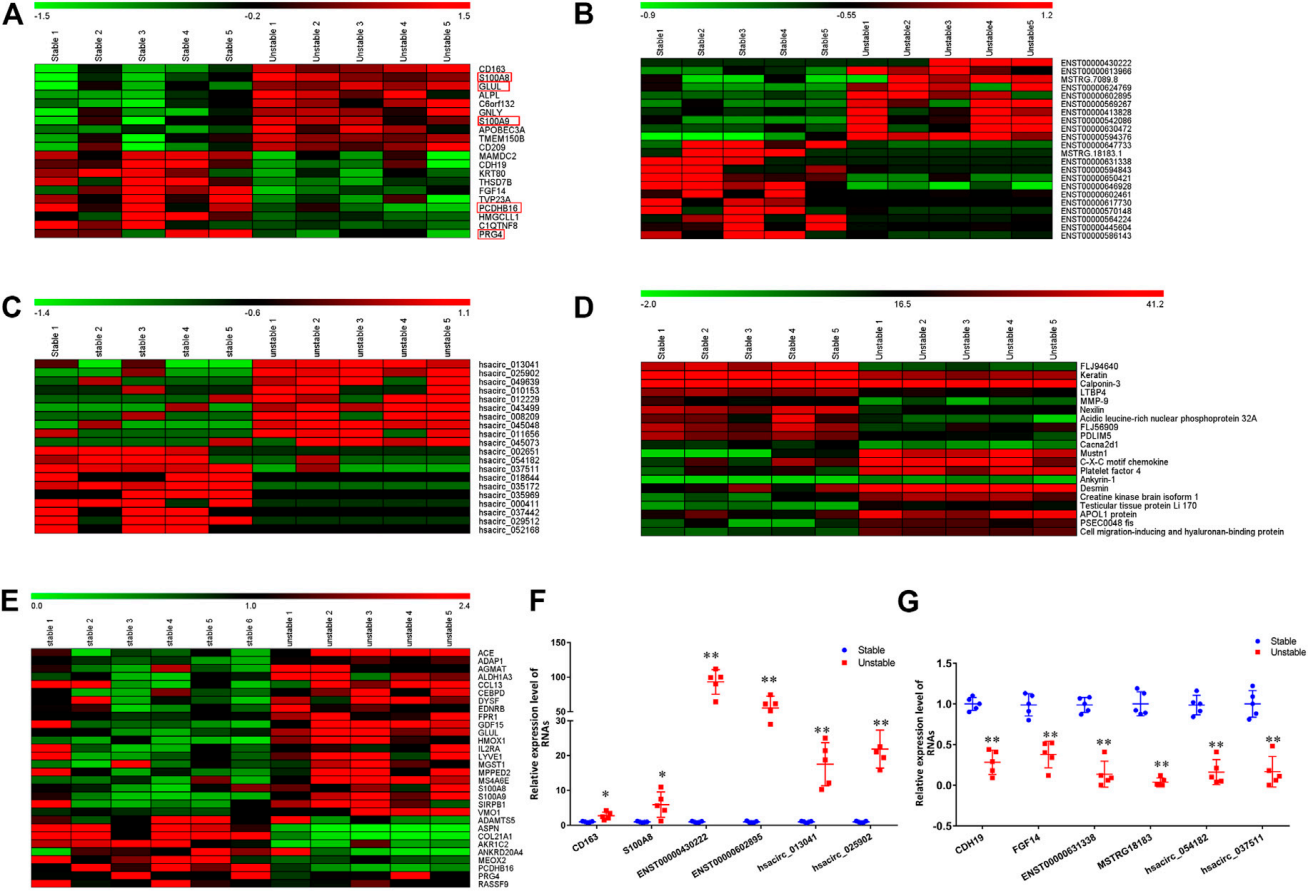
The hierarchical clustering heatmap of 20 DE mRNAs, lncRNAs, circRNAs, and proteins. A–D: The hierarchical clustering heatmap of mRNAs **(A)**, lncRNAs **(B)**, circRNA **(C)**, proteins **(D)**. **(E)**: The heatmap of overlapping genes between current study and GSE41571; **(F)**: the qPCR verification of 6 randomly selected upregulated mRNAs, lncRNAs, and circRNAs. **(G)**: the qPCR verification of 6 randomly selected downregulated mRNAs, lncRNAs, and circRNAs. The values (mean ± S.D. from 5 independent experiments) are relative to Stable group, which was set as 1. *p < 0.05, **p < 0.01. Red box: the overlapping genes between current study (A) and GSE41571 (E).

In the RNA-Seq analysis, more than 168 466 008 clean reads were identified with more than 96.57% been mapped to the reference genome. Totally, 20025 mRNAs, 31751 lncRNAs, and 12131 circRNAs were identified. Among these genes, 202 mRNAs, 488 lncRNAs, and 91 circRNAs were differentially expressed. In the 202 DE mRNAs, 125 were upregulated and 77 were downregulated in unstable plaques. The heatmap of 20 DE mRNAs were shown in Figure 1A. In unstable atherosclerotic plaques, 207 upregulated and 281 downregulated lncRNAs, 61 upregulated and 30 downregulated circRNAs were also been identified. The heatmaps of part of these differentially expressed lncRNAs and circRNAs were shown in Figures 1B,C. To verify the RNA-Seq results, the qPCR analysis was conducted for several DE mRNAs, lncRNAs, and circRNAs. The qPCR results were consistent with that of the RNA-Seq results (Figures 1F,G).

In the HPLC-MS/MS analysis, a total of 3,082 proteins in 23494 peptides were identified. Among these proteins, 148 were upregulated and 145 were downregulated in unstable plaques. The hierarchical clustering heatmap of 20 differentially expressed proteins were shown in Figure 1D.

### Comparison Analysis of the Present Transcriptome and Previously Reported Data from GSE41571.

To further verify our results and to figure out the key genes in the stability of atherosclerotic plaques, we analyzed the dataset GSE41571 downloaded from the GEO database. 1,595 downregulated and 750 upregulated genes were found in GSE41571. Among them, 30 genes were differentially expressed in both dataset GSE41571 and our RNA-Seq data, 42 genes were overlapped with our DEPs (Supplementary Table S2, Figure 1E). These overlapped genes were mainly related to cell adhesion, immune response, and inflammatory responses.

### The lncRNA-Targeted mRNA and circRNA-Originated Genes Identification

We screened the neighboring protein-coding genes of the 488 differentially expressed lncRNAs. 381 protein-coding genes were at the distance of 1 kb ∼ −1 kb to the corresponding 488 lncRNAs (Supplementary Table S3). These protein-coding genes were treated as the potential lncRNA-targeted genes. The corresponding lncRNAs were treated as *cis*-acting lncRNAs. The expression of these targeted genes might be regulated by the *cis*-acting lncRNAs.

We also analyzed the sequence of 91 differentially expressed circRNAs. All of these 91 circRNAs contained exonic sequences. These exonic sequences belong to 97 protein-coding genes. These 97 genes were considered to be the circRNA-originated genes (Supplementary Table S4).

The GO enrichment and KEGG pathway analysis of the DE mRNAs, lncRNA-targeted genes, circRNA-originated genes, and DEPs.

The GO and KEGG analysis was conducted to predict the functions of differentially expressed mRNA, the lncRNA-targeted genes, circRNA-originated genes, and the DEPs. The results were shown in Supplementary Table S5. The DE mRNAs were mainly enriched in the GO terms of extracellular region, RAGE receptor binding, defense response, and KEGG terms of neuron ligand-receptor interaction, and cytokine-receptor interaction. The lncRNA-targeted genes were enriched in the GO terms of intracellular membrane-bounded organelle, transcription regulator activity, transcription from RNA polymerase II promoter, and KEGG terms of the TNF signaling pathway. The circRNA-originated genes were enriched in the GO terms of basal cortex, malonyl-CoA decarboxylase activity, negative regulation of stress fiber assembly, and KEGG terms of cellular senescence and focal adhesion. The proteins were mainly related to ECM-receptor interaction, hematopoietic cell lineage, and phagosome.

### Protein-Protein Interaction Network and Clusters Analysis of Differentially Expressed Proteins

**FIGURE 2:**
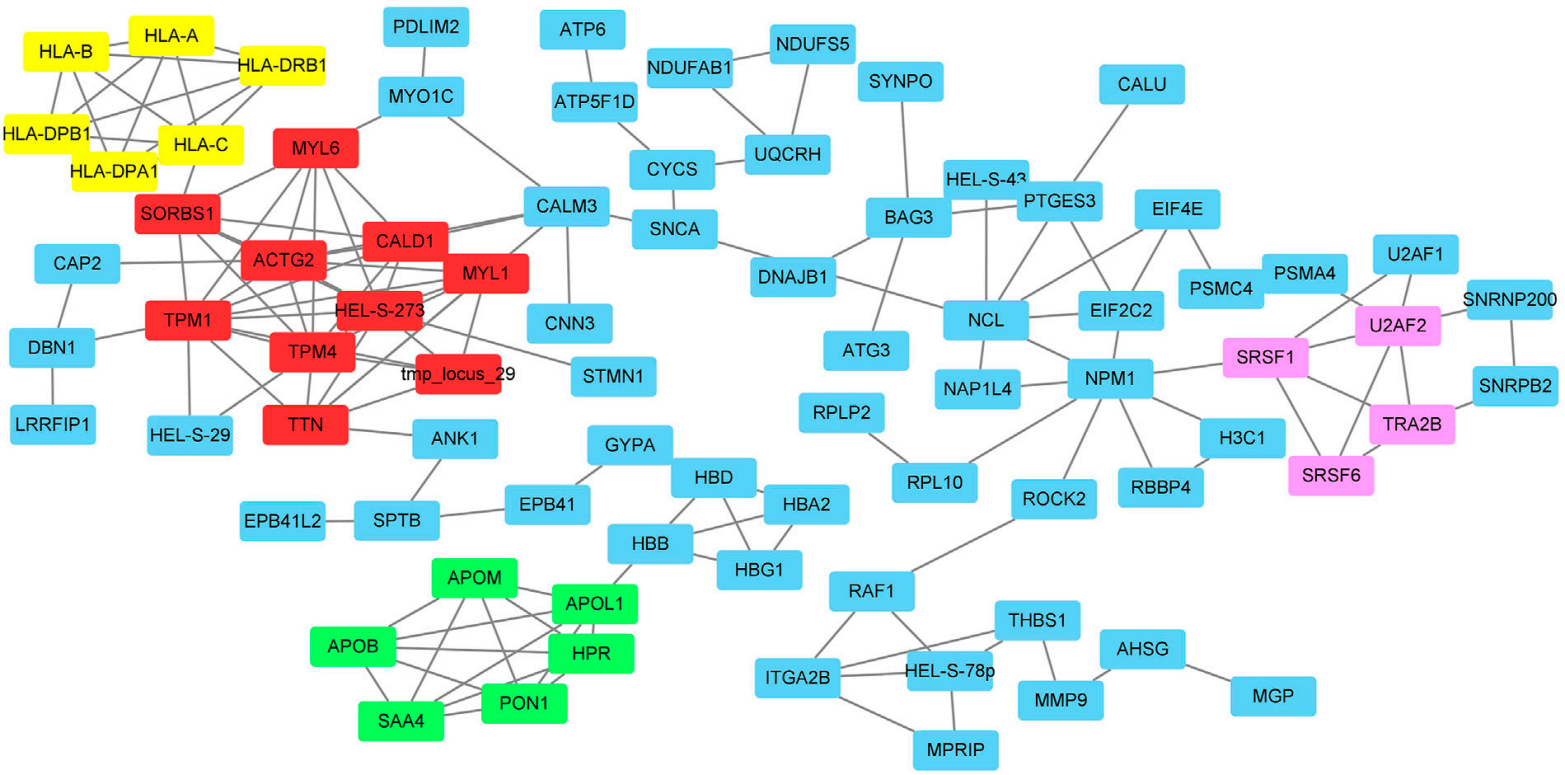
The protein-protein interactions of DEPs and the clusters identified by MCODE. Red box: cluster1; Green box: cluster 2; Yellow box: cluster 3; Pink box: cluster 4.

The protein-protein interaction network between the DEPs was analyzed by the online tool STRING and shown in Figure 2. In total, 195 nodes and 532 edges were identified. Four clusters with 24 genes were identified by the MCODE tool in the Cytoscape software (Figure 2 and Supplementary Table S6). The DEPs were clustered into 4 clusters which may be involved in the functions of smooth muscle contraction, metabolism and transportation of lipoproteins, immune system function, and mRNA splicing.

### Comparison Analysis of Transcriptome and Proteome

To analyze the consistency of transcripts and proteins, and to identify the key genes playing critical roles in the stability of atherosclerotic plaques, we compared the expression of differentially expressed mRNA and DEPs, the lncRNA-target genes and DEPs, as well as the circRNA-originated genes and DEPs. Surprisingly, only two DEPs (CD5L, S100A12) were overlapped with mRNA. Two proteins (CKB, CEMIP) were overlapped with the lncRNA-targeted genes, one protein (SH3GLB1) was overlapped with the cirRNA-originated gene. The expression levels of these mRNAs, related lncRNAs, related circRNA, and DEPs were shown in Supplementary Table S7. Both CD5L and S100A12 mRNAs and proteins were upregulated in the unstable plaques. The CKB and CEMIP proteins, as well as their related lncRNA, MSTRG.11455.17 and MSTRG.12845 were upregulated in unstable plaques. While the SH3GLB1 protein was upregulated, but its related circRNA, hsacirc_ooo411 was downregulated. These genes may play critical roles in the stability of atherosclerotic plaques.

### LncRNA (circRNA)-miRNA-mRNA Network Analysis

**FIGURE 3:**
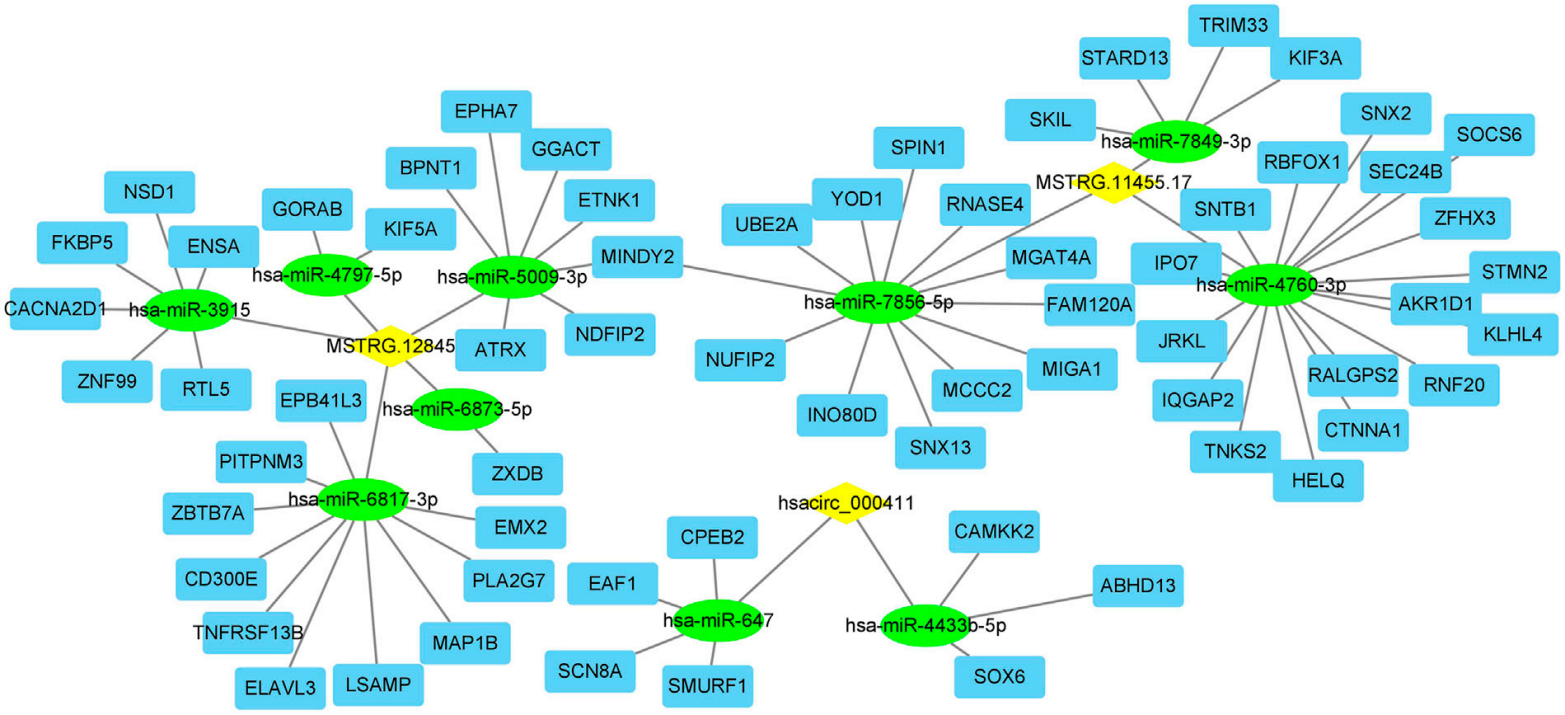
The lncRNA (or circRNA)-miRNA-mRNA interactions network. Yellow diamond: lncRNA or circRNA; green circle: miRNA; blue square: mRNA.

In *LncRNA (circRNA)-miRNA-mRNA Network Analysis*, two lncRNAs (MSTRG.11455.17, MSTRG.12845) and one circRNA (hsacirc_000411) were identified to interact with DEPs. To further explore the functions of MSTRG.11455.17, MSTRG.12845, and hascirc_000411, we analyzed the related miRNA, and the subsequent miRNA targeted mRNAs. The lncRNA (circRNA)-miRNA-mRNA network was shown in Figure 3. The lncRNA MSTRG.11455.17 was predicted to bind miR-7849, miR-7856, and miR-4760, which may affect the functions of subsequent 33 genes. LncRNA MSTRG.12845 was predicted to bind miR-4797, miR-3915, miR-5009, miR-6873, and miR-6817, which may affect subsequent 26 genes. Hsacirc-000411 was predicted to bind miR-647 and miR-4433b, which may affect subsequent 7 genes (Figure 3).

## Discussion

The present study screened the transcripts and proteins in human stable and unstable atherosclerotic plaques. A total of 202 mRNAs, 488 lncRNAs, 91 circRNAs, and 293 DEPs were identified to be differentially expressed between stable and unstable atherosclerotic plaques. The 488 lncRNAs might affect the production of 381 protein-coding genes by *cis*-acting regulations. Sequence analysis indicated the 91 circRNAs were originated from 97 protein-coding genes.

To figure out the functions of these DE mRNAs, lncRNAs, circRNAs, and proteins, we conducted the GO enrichment and KEGG pathway analysis, as well as the PPI network. The enrichment and pathway analysis indicated these differentially expressed RNAs and proteins were involved in the cellular response to stress or stimulus, regulated the gene transcription through ways such as histone acetyltransferase binding, spliceosome, and dihydropteridine reductase activity. In the atherosclerotic plaque stability, the following functions may play critical roles: the immune response (RAGE receptor binding, cytokine-cytokine receptor interactions, ECM-receptor interaction, phagosome, B cell receptor signaling pathway, cGMP-PKG signaling pathway, antigen processing and presentation), nervous system functions (neuroactive ligand-receptor interactions, cholinergic synapse, neurotrophin signaling pathway), hematologic activities (hematopoietic cell lineage, coagulation cascades), and endocrine system (cortisol synthesis and secretion, insulin secretion). The PPI and MCODE analysis of DEPs discovered 4 clusters relating to the function of smooth muscle contraction, insulin function, lipid metabolism, immune system, and gene expression (Supplementary Table S6). These results indicated that the immune response, endocrine system, metabolisms are major functional alterations between stable and unstable atherosclerotic plaques.

Since the previous microarray dataset GSE41571 analyzed the gene expression profiles in the macrophage-rich regions of stable and unstable atherosclerotic plaques, we compared our data with the GSE41571 data. 30 genes and 42 proteins were found to be differentially expressed in both our data and GSE41571. These 72 genes and proteins are mainly related to cell adhesion, immune response, and inflammatory responses, which were consistent with our GO and KEGG analysis.

To further screen out the key genes which may play critical roles in the stability of atherosclerosis, we analyzed the consistency of transcriptome and proteome in the present study. Surprisingly, only a few genes were screened out. They are CD5L, S100A12, CKB (target gene of lncRNA MSTRG.11455.17), CEMIP (target gene of lncRNA MSTRG.12845), and SH3GLB1 (originated gene of hsacirc_000411). CD5L and S100A12 were upregulated in unstable plaques at both mRNA and protein levels. CD5L encodes the secreted glycoprotein antigen protein CD5, which is involved in the inflammatory response. It is primarily expressed in macrophages and promotes M2 macrophage polarization, promotes anti-inflammation in response to TLR activation (Sanjurjo et al., 2015, 2018). S100A12 is a member of the S100 protein family. It binds to RAGE and activates the downstream pro-inflammatory signals, such as NF-kB and ROS (Xiao et al., 2020). S100A12 is involved in the pathogenesis of atherosclerosis through the S100A12-CD36 axis (Farokhzadian et al., 2019). CKB and CEMIP are the targets of two novel lncRNAs MSTRG.11455.17 and MSTRG.12845, respectively. CKB encodes protein creatine kinase B, which plays a role in energetic hemostasis in ischemic and inflammatory disorders (Kitzenberg et al., 2016). CEMIP encodes the cell migration-inducing and hyaluronan-binding protein, which regulates epithelial-mesenchymal transition (EMT), tumor cell growth and migration (Li et al., 2017). In atherosclerosis, CEMIP was reported to regulate the proliferation and migration of vascular smooth muscle cells (Xue et al., 2020). SH3GLB1 gene encodes the endophilin-B1 or Bif-1 protein, which is implicated in the apoptotic and autophagic pathways (Takahashi et al., 2013). However, its effects on atherosclerosis are still unknown. Therefore, the above five genes, and their correlated lncRNAs (MSTRG.11455.17, MSTRG.12845) and circRNA (circ_000411) may play critical roles in the stability of atherosclerotic plaques through inflammation, cell growth or migration.

Competitively endogenous RNA (ceRNA) is another important mechanism of the functions of non-coding RNAs. ceRNAs regulate other RNA transcripts by competing for shared microRNAs (miRNAs) (Salmena et al., 2011). Based on the sequence of MSTRG.11455.17, MSTRG.12845, and circ_000411, we found 10 miRNAs that may bind to them, indicating their ceRNA potential.

In our present study, a whole atherosclerotic plaque was collected through method of CEA, and was used for detection. Atherosclerotic plaques are comprised of many different kinds of cells, such as foam cells, macrophages, smooth muscle cells. Different cell types are with different gene expression patterns. This is a major limitation in our present analysis, which might decrease the efficacy of screening out more differentially expressed genes. Single-cell RNA-Seq is needed in future studies to distinguish the gene expression pattern in different cells types between stable and unstable plaques.

In summary, our study screened the transcription and protein profiles in human stable and unstable atherosclerotic plaques by RNA-Seq and LC-MS/MS, analyzed the functions and pathways of differentially expressed RNAs and proteins, identified a few key genes and noncoding RNAs. The results may provide new knowledge on understanding the stability of atherosclerotic plaques.

## Data Availability

The transcriptome datasets PRJNA752896 for this study can be found in the Sequence Read Archive (SRA) of National Library of Medicine [https://submit.ncbi.nlm.nih.gov/subs/bioproject/]. The proteome datasets were submitted to the iProX (https://www.iprox.cn/), with the Project ID of IPX0003457000.
