# Supplementary material for: Transcriptomic and Proteomic Profiling of Human Stable and Unstable Carotid Atherosclerotic Plaques
Source: Front Genet. 2021 Nov 4;12:755507. doi: 10.3389/fgene.2021.755507 (PMC8599967; doi:10.3389/fgene.2021.755507)
Supplement: Supplementary file 1 [file Table1.docx]

**Table 1 Characteristics of included patients**

| Characteristics | Patients with unstable plaques  (n=5) | Patients with stable plaques  (n = 5) | *P*-value |
| --- | --- | --- | --- |
| Age (years) | 64.04±10.36 | 55.72±4.76 | 0.237 |
| Male (%) | 80.00(4) | 100.00(5) | - |
| Body weight index | 24.33±3.28 | 23.09±2.16 | 0.896 |
| Smoke (%) | 80.00(4) | 60.00(3) | - |
| Alcohol (%) | 60.00(3) | 60.00(3) | - |
| Antihypertensives (%) | 40.00(2) | 40.00(2) | - |
| Antihyperlipidemic drugs (%) | 40.00(2) | 0 | - |
| Antiplatelet drugs(%) | 40.00(2) | 0 | - |
| TG | 3.49±1.11 | 3.59±1.45 | 0.437 |
| HDL | 0.92±0.14 | 0.81±0.11 | 0.357 |
| LDL | 2.46±0.23 | 1.89±0.14 | 0.425 |
| TC | 1.45±0.34 | 1.02±0.21 | 0.451 |

TG: triglycerides; HDL: high density lipoprotein; LDL: low density lipoprotein; TC: total cholesterol;
