# Supplementary material for: Transcriptomic and Proteomic Profiling of Human Stable and Unstable Carotid Atherosclerotic Plaques
Source: Front Genet. 2021 Nov 4;12:755507. doi: 10.3389/fgene.2021.755507 (PMC8599967; doi:10.3389/fgene.2021.755507)
Supplement: Supplementary file 2 [file Table7.docx]

Table 7 The fold change and *P*-value of overlapped genes or proteins

| **Gene name** | **Related lncRNA/**  **circRNA** | **lncRNA/**  **circRNA**  **(FC)** | **LncRNA/**  **circRNA**  **P-value** | **mRNA**  **(FC)** | **mRNA**  ***P*-value** | **Protein**  **(FC)** | **Protein**  **P-value** |
| --- | --- | --- | --- | --- | --- | --- | --- |
| CD5L | - | - | - | 13.26 | < 0.05 | 2.06 | < 0.01 |
| S100A12 | - | - | - | 5.81 | < 0.01 | 6.36 | < 0.01 |
| CKB | MSTRG.11455.17 | infinite | < 0.01 | - | - | 8.22 | < 0.01 |
| CEMIP | MSTRG.12845 | 2.011 | < 0.01 | - | - | 3.03 | < 0.01 |
| SH3GLB1 | hsacirc_000411 | -infinite | < 0.05 | - | - | 2.35 | < 0.05 |
