# Supplementary material for: Transcriptomic and Proteomic Profiling of Human Stable and Unstable Carotid Atherosclerotic Plaques
Source: Front Genet. 2021 Nov 4;12:755507. doi: 10.3389/fgene.2021.755507 (PMC8599967; doi:10.3389/fgene.2021.755507)
Supplement: Supplementary file 3 [file Table2.docx]

Table 2 The overlapped genes identified from GSE41571 and from the present DE mRNAs and DEPs

| **Overlap** | **Genes** |
| --- | --- |
| GSE41571 and present DEGs (30) | ACE, ADAP1, AGMAT, ALDH1A3, CCL13, CEBPD, DYSF, EDNRB, FPR1, GDF15, GLUL, HMOX1, IL2RA, LYVE1, MGST1, MPPED2, MS4A6E, S100A8, S100A9, SIRPB1, VMO1, ADAMTS5, ASPN, COL21A1, AKR1C2, ANKRD20A4, MEOX2, PCDHB16, PRG4, RASSF9 |
| GSE41571 and present DEPs (42) | APOC4-APOC2, C5AR1, CALM3, CCL18, CTSG, FCGRT, HBA2, HBB, HLA-B, HLA-DPA1, MGAT1, MGAT4B, MMP12, PNPO, AKR1C3, CACNA2D1, CALD1, CAP2, CEMIP, CFHR2, CNN3, EDIL3, HAPLN1, HMCN1, IGFBP7, KIAA1671, LOXL4, LRRFIP1, MFGE8, MGP, NEXN, NUCKS1, PPP1R14A, SORBS1, SPARC, SYNPO, THBS1, TNC, TNXB, TPM1, TPM4, WISP2 |
