# Supplementary material for: Transcriptomic and Proteomic Profiling of Human Stable and Unstable Carotid Atherosclerotic Plaques
Source: Front Genet. 2021 Nov 4;12:755507. doi: 10.3389/fgene.2021.755507 (PMC8599967; doi:10.3389/fgene.2021.755507)
Supplement: Supplementary file 4 [file Table5.docx]

**Table 5 The GO and KEGG enrichment of differentially expressed RNAs and DEPs**

| **Category** | **Enriched Terms** | **Number** | ***P*-value** |
| --- | --- | --- | --- |
| GO-Biological Process (mRNA) | Defense response | 42 | 9.5E-09 |
|  | Response to external stimulus | 51 | 7.9E-08 |
|  | Anatomical structure development | 92 | 5.5E-07 |
|  | Response to stress | 69 | 5.9E-07 |
|  | Developmental process | 95 | 1.7E-06 |
|  | System development | 77 | 5.7E-06 |
| GO-Cellular Components  (mRNA) | Extracellular region | 74 | 1.8E-07 |
|  | Extracellular region part | 58 | 0.000019 |
|  | Extracellular space | 54 | 0.000079 |
|  | Extracellular matrix | 12 | 0.00012 |
|  | Intrinsic component of plasma membrane | 34 | 0.00016 |
| GO-Molecular Function  (mRNA) | RAGE receptor binding | 4 | 3.2E-06 |
|  | Organic acid binding | 10 | 0.000065 |
|  | Receptor ligand activity | 15 | 0.00013 |
|  | Carboxylic acid binding | 9 | 0.00022 |
|  | Growth factor activity | 8 | 0.00029 |
| KEGG pathway  (mRNA) | Neuroactive ligand-receptor interaction | 10 | 0.00119 |
|  | Complement and coagulation cascades | 4 | 0.00891 |
|  | Cytokine-cytokine receptor interaction | 8 | 0.01029 |
|  | Cocaine addiction | 3 | 0.01394 |
|  | MicroRNAs in cancer | 5 | 0.01527 |
| GO-Biological Process  (lncRNA) | Transcription from RNA polymerase II promoter | 83 | 0.000013 |
|  | Nucleic acid-templated transcription | 103 | 0.000015 |
|  | DNA-templated transcription | 102 | 0.000015 |
|  | RNA biosynthetic process | 103 | 0.000018 |
|  | Aromatic compound biosynthetic process | 112 | 0.000032 |
| GO-Cellular Components  (lncRNA) | Intracellular membrane-bounded organelle | 242 | 0.00013 |
|  | Membrane-bounded organelle | 267 | 0.00019 |
|  | Organelle | 285 | 0.0003 |
|  | Nucleus | 171 | 0.00055 |
|  | Nuclear lumen | 104 | 0.00112 |
| GO-Molecular Function  (lncRNA) | Transcription regulator activity | 62 | 0.00015 |
|  | transcriptional activator activity | 20 | 0.00018 |
|  | Regulatory region DNA binding | 34 | 0.00029 |
|  | Transcription regulatory region DNA binding | 34 | 0.00029 |
|  | Regulatory region nucleic acid binding | 34 | 0.0003 |
| KEGG pathway  (lncRNA) | Vasopressin-regulated water reabsorption | 5 | 0.00126 |
|  | Prostate cancer | 7 | 0.00190 |
|  | Cholinergic synapse | 7 | 0.00479 |
|  | Insulin secretion | 6 | 0.00491 |
|  | Colorectal cancer | 6 | 0.00520 |
| GO-Biological Process  (circRNA) | Negative regulation of stress fiber assembly | 3 | 0.00014 |
|  | Negative regulation of wound healing | 2 | 0.00014 |
|  | Negative regulation of actin filament bundle assembly | 3 | 0.00021 |
|  | Macromolecule localization | 29 | 0.00023 |
|  | Regulation of basement membrane organization | 2 | 0.00023 |
| GO-Cellular Components  (circRNA) | Basal cortex | 2 | 0.00022 |
|  | Intracellular organelle part | 57 | 0.00246 |
|  | Non-membrane-bounded organelle | 32 | 0.0026 |
|  | Protein complex | 24 | 0.00275 |
|  | Postsynapse | 9 | 0.00278 |
| GO-Molecular Function  (circRNA) | Malonyl-CoA decarboxylase activity | 2 | 0.000024 |
|  | Histone acetyltransferase binding | 3 | 0.00038 |
|  | 3',5'-cyclic-GMP phosphodiesterase activity | 2 | 0.00346 |
|  | 6,7-dihydropteridine reductase activity | 1 | 0.0049 |
|  | Beta-mannosidase activity | 1 | 0.0049 |
| KEGG pathway  (circRNA) | B cell receptor signaling pathway | 4 | 0.000354996 |
|  | cGMP-PKG signaling pathway | 5 | 0.001159661 |
|  | Neurotrophin signaling pathway | 4 | 0.002716875 |
|  | Cortisol synthesis and secretion | 3 | 0.003596882 |
|  | HTLV-I infection | 5 | 0.004057118 |
| GO-Biological Process  (DEPs) | Response to stress | 162 | 1.37E-79 |
|  | Positive regulation of biological process | 159 | 6.05E-66 |
|  | Response to stimulus | 135 | 1.39E-63 |
|  | Localization | 192 | 1.33E-61 |
|  | Response to chemical | 132 | 1.71E-57 |
| GO-Cellular Components  (DEPs) | Extracellular region part | 172 | 6.69E-125 |
|  | Extracellular region | 189 | 2.23E-123 |
|  | Membrane-bounded vesicle | 152 | 8E-114 |
|  | Vesicle | 158 | 6.06E-108 |
|  | Extracellular organelle | 130 | 6.06E-108 |
| GO-Molecular Function  (DEPs) | Protein binding | 223 | 1.63E-80 |
|  | Antigen binding | 37 | 8.17E-37 |
|  | Binding | 273 | 7.3E-36 |
|  | Identical protein binding | 43 | 1.24E-17 |
|  | Poly(A) RNA binding | 34 | 1.95E-17 |
| KEGG pathway  (DEPs) | ECM-receptor interaction | 10 | 0.0000296 |
|  | Hematopoietic cell lineage | 10 | 0.0000549 |
|  | Phagosome | 13 | 0.000108 |
|  | Spliceosome | 11 | 0.000443 |
|  | Antigen processing and presentation | 8 | 0.000627 |
