# Supplementary material for: Transcriptomic and Proteomic Profiling of Human Stable and Unstable Carotid Atherosclerotic Plaques
Source: Front Genet. 2021 Nov 4;12:755507. doi: 10.3389/fgene.2021.755507 (PMC8599967; doi:10.3389/fgene.2021.755507)
Supplement: Supplementary file 5 [file Table3.docx]

Table 3 lncRNA targeted genes

| **lncRNAGeneID** | **mRNAGeneID** | **mRNAGeneName** |
| --- | --- | --- |
| ENSG00000128254 | ENSG00000285404 | Z82190.2 |
| ENSG00000128254 | ENSG00000128245 | YWHAH |
| ENSG00000166770 | ENSG00000198046 | ZNF667 |
| ENSG00000172965 | ENSG00000153094 | BCL2L11 |
| ENSG00000172965 | ENSG00000153093 | ACOXL |
| ENSG00000174171 | ENSG00000137877 | SPTBN5 |
| ENSG00000174171 | ENSG00000103966 | EHD4 |
| ENSG00000178803 | ENSG00000100024 | UPB1 |
| ENSG00000178803 | ENSG00000128271 | ADORA2A |
| ENSG00000178803 | ENSG00000258555 | SPECC1L-ADORA2A |
| ENSG00000179406 | ENSG00000169902 | TPST1 |
| ENSG00000179818 | ENSG00000244617 | ASPRV1 |
| ENSG00000179818 | ENSG00000169564 | PCBP1 |
| ENSG00000182057 | ENSG00000100207 | TCF20 |
| ENSG00000189223 | ENSG00000125618 | PAX8 |
| ENSG00000196295 | ENSG00000281039 | AC005154.5 |
| ENSG00000203930 | ENSG00000184258 | CDR1 |
| ENSG00000204380 | ENSG00000144283 | PKP4 |
| ENSG00000204706 | ENSG00000165072 | MAMDC2 |
| ENSG00000212694 | ENSG00000139725 | RHOF |
| ENSG00000216895 | ENSG00000184863 | RBM33 |
| ENSG00000222020 | ENSG00000068024 | HDAC4 |
| ENSG00000224661 | ENSG00000151360 | ALLC |
| ENSG00000224661 | ENSG00000214866 | DCDC2C |
| ENSG00000225138 | ENSG00000066230 | SLC9A3 |
| ENSG00000225206 | ENSG00000188641 | DPYD |
| ENSG00000225342 | ENSG00000188906 | LRRK2 |
| ENSG00000225465 | ENSG00000128250 | RFPL1 |
| ENSG00000225746 | ENSG00000254656 | RTL1 |
| ENSG00000225914 | ENSG00000204296 | TSBP1 |
| ENSG00000225914 | ENSG00000204290 | BTNL2 |
| ENSG00000227540 | ENSG00000156042 | CFAP70 |
| ENSG00000228203 | ENSG00000151692 | RNF144A |
| ENSG00000228242 | ENSG00000154767 | XPC |
| ENSG00000228242 | ENSG00000268279 | AC090004.1 |
| ENSG00000228956 | ENSG00000182568 | SATB1 |
| ENSG00000229124 | ENSG00000026025 | VIM |
| ENSG00000229368 | ENSG00000167323 | STIM1 |
| ENSG00000229950 | ENSG00000137203 | TFAP2A |
| ENSG00000229980 | ENSG00000141232 | TOB1 |
| ENSG00000231160 | ENSG00000109787 | KLF3 |
| ENSG00000231607 | ENSG00000204977 | TRIM13 |
| ENSG00000231607 | ENSG00000198553 | KCNRG |
| ENSG00000231871 | ENSG00000198700 | IPO9 |
| ENSG00000231871 | ENSG00000134369 | NAV1 |
| ENSG00000232021 | ENSG00000138795 | LEF1 |
| ENSG00000232295 | ENSG00000119900 | OGFRL1 |
| ENSG00000232788 | ENSG00000091409 | ITGA6 |
| ENSG00000232807 | ENSG00000134452 | FBH1 |
| ENSG00000232807 | ENSG00000134470 | IL15RA |
| ENSG00000232973 | ENSG00000138061 | CYP1B1 |
| ENSG00000232995 | ENSG00000143248 | RGS5 |
| ENSG00000232995 | ENSG00000143228 | NUF2 |
| ENSG00000233230 | ENSG00000138081 | FBXO11 |
| ENSG00000233766 | ENSG00000168497 | CAVIN2 |
| ENSG00000233766 | ENSG00000144339 | TMEFF2 |
| ENSG00000233937 | ENSG00000204628 | RACK1 |
| ENSG00000233937 | ENSG00000183718 | TRIM52 |
| ENSG00000234456 | ENSG00000187391 | MAGI2 |
| ENSG00000234741 | ENSG00000185278 | ZBTB37 |
| ENSG00000234741 | ENSG00000117593 | DARS2 |
| ENSG00000234912 | ENSG00000129657 | SEC14L1 |
| ENSG00000235092 | ENSG00000115738 | ID2 |
| ENSG00000235257 | ENSG00000144668 | ITGA9 |
| ENSG00000236859 | ENSG00000155438 | NIFK |
| ENSG00000237125 | ENSG00000164107 | HAND2 |
| ENSG00000237181 | ENSG00000188191 | PRKAR1B |
| ENSG00000237298 | ENSG00000155657 | TTN |
| ENSG00000239665 | ENSG00000151474 | FRMD4A |
| ENSG00000239665 | ENSG00000282246 | AL157392.5 |
| ENSG00000240405 | ENSG00000242120 | MDFIC2 |
| ENSG00000241769 | ENSG00000241489 | AC244197.3 |
| ENSG00000241769 | ENSG00000197620 | CXorf40A |
| ENSG00000242082 | ENSG00000100191 | SLC5A4 |
| ENSG00000242086 | ENSG00000176945 | MUC20 |
| ENSG00000243069 | ENSG00000114790 | ARHGEF26 |
| ENSG00000244675 | ENSG00000133657 | ATP13A3 |
| ENSG00000245105 | ENSG00000175899 | A2M |
| ENSG00000245573 | ENSG00000176697 | BDNF |
| ENSG00000246695 | ENSG00000123094 | RASSF8 |
| ENSG00000247498 | ENSG00000013583 | HEBP1 |
| ENSG00000247498 | ENSG00000111291 | GPRC5D |
| ENSG00000247572 | ENSG00000131730 | CKMT2 |
| ENSG00000247572 | ENSG00000113319 | RASGRF2 |
| ENSG00000247809 | ENSG00000185551 | NR2F2 |
| ENSG00000247934 | ENSG00000123106 | CCDC91 |
| ENSG00000248101 | ENSG00000239382 | ALKBH6 |
| ENSG00000248101 | ENSG00000181392 | SYNE4 |
| ENSG00000248323 | ENSG00000164199 | ADGRV1 |
| ENSG00000248734 | ENSG00000164307 | ERAP1 |
| ENSG00000248932 | ENSG00000163864 | NMNAT3 |
| ENSG00000248932 | ENSG00000114113 | RBP2 |
| ENSG00000248932 | ENSG00000114115 | RBP1 |
| ENSG00000249042 | ENSG00000152380 | FAM151B |
| ENSG00000249042 | ENSG00000039319 | ZFYVE16 |
| ENSG00000250069 | ENSG00000120306 | CYSTM1 |
| ENSG00000250280 | ENSG00000174099 | MSRB3 |
| ENSG00000250802 | ENSG00000284762 | AC022414.1 |
| ENSG00000250802 | ENSG00000132846 | ZBED3 |
| ENSG00000250802 | ENSG00000285000 | AC008581.2 |
| ENSG00000253438 | ENSG00000212993 | POU5F1B |
| ENSG00000253438 | ENSG00000168672 | FAM84B |
| ENSG00000254473 | ENSG00000135018 | UBQLN1 |
| ENSG00000255036 | ENSG00000197816 | CCDC180 |
| ENSG00000255248 | ENSG00000259571 | BLID |
| ENSG00000255389 | ENSG00000056972 | TRAF3IP2 |
| ENSG00000256482 | ENSG00000060982 | BCAT1 |
| ENSG00000256628 | ENSG00000066422 | ZBTB11 |
| ENSG00000257467 | ENSG00000139220 | PPFIA2 |
| ENSG00000258325 | ENSG00000111203 | ITFG2 |
| ENSG00000258325 | ENSG00000004478 | FKBP4 |
| ENSG00000258376 | ENSG00000100767 | PAPLN |
| ENSG00000258738 | ENSG00000198604 | BAZ1A |
| ENSG00000258940 | ENSG00000150527 | MIA2 |
| ENSG00000259583 | ENSG00000184254 | ALDH1A3 |
| ENSG00000259649 | ENSG00000103888 | CEMIP |
| ENSG00000259673 | ENSG00000189227 | C15orf61 |
| ENSG00000259673 | ENSG00000103599 | IQCH |
| ENSG00000259940 | ENSG00000155666 | KDM8 |
| ENSG00000260136 | ENSG00000168434 | COG7 |
| ENSG00000260267 | ENSG00000140691 | ARMC5 |
| ENSG00000260388 | ENSG00000136143 | SUCLA2 |
| ENSG00000260470 | ENSG00000123415 | SMUG1 |
| ENSG00000260630 | ENSG00000185669 | SNAI3 |
| ENSG00000260816 | ENSG00000186153 | WWOX |
| ENSG00000261183 | ENSG00000166145 | SPINT1 |
| ENSG00000261215 | ENSG00000187186 | AL162231.1 |
| ENSG00000261215 | ENSG00000213927 | CCL27 |
| ENSG00000261269 | ENSG00000178175 | ZNF366 |
| ENSG00000261409 | ENSG00000260548 | AL035425.2 |
| ENSG00000261596 | ENSG00000197006 | METTL9 |
| ENSG00000261801 | ENSG00000129038 | LOXL1 |
| ENSG00000261959 | ENSG00000108823 | SGCA |
| ENSG00000261971 | ENSG00000008516 | MMP25 |
| ENSG00000261997 | ENSG00000087253 | LPCAT2 |
| ENSG00000262312 | ENSG00000167984 | NLRC3 |
| ENSG00000262413 | ENSG00000141522 | ARHGDIA |
| ENSG00000263072 | ENSG00000085644 | ZNF213 |
| ENSG00000263072 | ENSG00000122386 | ZNF205 |
| ENSG00000263244 | ENSG00000182831 | C16orf72 |
| ENSG00000263400 | ENSG00000187824 | TMEM220 |
| ENSG00000263400 | ENSG00000263429 | TMEM238L |
| ENSG00000263843 | ENSG00000125454 | SLC25A19 |
| ENSG00000264754 | ENSG00000154217 | PITPNC1 |
| ENSG00000265257 | ENSG00000101745 | ANKRD12 |
| ENSG00000265257 | ENSG00000178127 | NDUFV2 |
| ENSG00000266923 | ENSG00000205057 | CLLU1OS |
| ENSG00000267058 | ENSG00000176222 | ZNF404 |
| ENSG00000267272 | ENSG00000267561 | AC093155.3 |
| ENSG00000267504 | ENSG00000049759 | NEDD4L |
| ENSG00000267749 | ENSG00000176533 | GNG7 |
| ENSG00000268858 | ENSG00000183260 | ABHD16B |
| ENSG00000269293 | ENSG00000196812 | ZSCAN16 |
| ENSG00000269397 | ENSG00000213967 | ZNF726 |
| ENSG00000269918 | ENSG00000171044 | XKR6 |
| ENSG00000269937 | ENSG00000140992 | PDPK1 |
| ENSG00000269949 | ENSG00000084093 | REST |
| ENSG00000270956 | ENSG00000155657 | TTN |
| ENSG00000271122 | ENSG00000122557 | HERPUD2 |
| ENSG00000271147 | ENSG00000286237 | ARMCX5-GPRASP2 |
| ENSG00000271147 | ENSG00000158301 | GPRASP2 |
| ENSG00000271147 | ENSG00000125962 | ARMCX5 |
| ENSG00000271147 | ENSG00000198932 | GPRASP1 |
| ENSG00000271335 | ENSG00000108100 | CCNY |
| ENSG00000271855 | ENSG00000151694 | ADAM17 |
| ENSG00000271980 | ENSG00000150753 | CCT5 |
| ENSG00000272054 | ENSG00000115816 | CEBPZ |
| ENSG00000272054 | ENSG00000218739 | CEBPZOS |
| ENSG00000272269 | ENSG00000124789 | NUP153 |
| ENSG00000272505 | ENSG00000253649 | PRSS51 |
| ENSG00000272787 | ENSG00000099974 | DDTL |
| ENSG00000273080 | ENSG00000132305 | IMMT |
| ENSG00000273186 | ENSG00000167123 | CERCAM |
| ENSG00000273247 | ENSG00000164134 | NAA15 |
| ENSG00000273247 | ENSG00000172007 | RAB33B |
| ENSG00000273328 | ENSG00000144648 | ACKR2 |
| ENSG00000273328 | ENSG00000180432 | CYP8B1 |
| ENSG00000273328 | ENSG00000273291 | AC092042.3 |
| ENSG00000273328 | ENSG00000240747 | KRBOX1 |
| ENSG00000273328 | ENSG00000182983 | ZNF662 |
| ENSG00000273729 | ENSG00000119669 | IRF2BPL |
| ENSG00000274227 | ENSG00000089248 | ERP29 |
| ENSG00000274964 | ENSG00000151746 | BICD1 |
| ENSG00000275457 | ENSG00000088930 | XRN2 |
| ENSG00000275576 | ENSG00000101337 | TM9SF4 |
| ENSG00000275807 | ENSG00000168488 | ATXN2L |
| ENSG00000276166 | ENSG00000140859 | KIFC3 |
| ENSG00000276278 | ENSG00000176371 | ZSCAN2 |
| ENSG00000276564 | ENSG00000186260 | MRTFB |
| ENSG00000277715 | ENSG00000136026 | CKAP4 |
| ENSG00000278126 | ENSG00000184271 | AC139768.1 |
| ENSG00000278419 | ENSG00000067057 | PFKP |
| ENSG00000278419 | ENSG00000107959 | PITRM1 |
| ENSG00000278831 | ENSG00000258539 | AC068896.1 |
| ENSG00000278831 | ENSG00000189319 | FAM53B |
| ENSG00000278985 | ENSG00000286221 | AC009070.1 |
| ENSG00000278985 | ENSG00000103121 | CMC2 |
| ENSG00000279035 | ENSG00000125458 | NT5C |
| ENSG00000279066 | ENSG00000169660 | HEXD |
| ENSG00000279118 | ENSG00000155324 | GRAMD2B |
| ENSG00000279133 | ENSG00000108510 | MED13 |
| ENSG00000279275 | ENSG00000168675 | LDLRAD4 |
| ENSG00000279355 | ENSG00000026652 | AGPAT4 |
| ENSG00000279377 | ENSG00000160321 | ZNF208 |
| ENSG00000279500 | ENSG00000139370 | SLC15A4 |
| ENSG00000279539 | ENSG00000268643 | AC006486.1 |
| ENSG00000279568 | ENSG00000140992 | PDPK1 |
| ENSG00000279569 | ENSG00000132613 | MTSS1L |
| ENSG00000279573 | ENSG00000171634 | BPTF |
| ENSG00000279608 | ENSG00000174038 | C9orf131 |
| ENSG00000279620 | ENSG00000103489 | XYLT1 |
| ENSG00000279649 | ENSG00000103061 | SLC7A6OS |
| ENSG00000279649 | ENSG00000103064 | SLC7A6 |
| ENSG00000279660 | ENSG00000109099 | PMP22 |
| ENSG00000279662 | ENSG00000166669 | ATF7IP2 |
| ENSG00000279811 | ENSG00000197971 | MBP |
| ENSG00000279932 | ENSG00000003989 | SLC7A2 |
| ENSG00000279970 | ENSG00000140479 | PCSK6 |
| ENSG00000280035 | ENSG00000022567 | SLC45A4 |
| ENSG00000280036 | ENSG00000128908 | INO80 |
| ENSG00000280120 | ENSG00000051825 | MPHOSPH9 |
| ENSG00000280239 | ENSG00000167671 | UBXN6 |
| ENSG00000280401 | ENSG00000156574 | NODAL |
| ENSG00000280798 | ENSG00000176102 | CSTF3 |
| ENSG00000280798 | ENSG00000176148 | TCP11L1 |
| ENSG00000282386 | ENSG00000143570 | SLC39A1 |
| ENSG00000282386 | ENSG00000285779 | AL358472.7 |
| ENSG00000283684 | ENSG00000284337 | AC013271.1 |
| ENSG00000284959 | ENSG00000165409 | TSHR |
| ENSG00000285184 | ENSG00000136631 | VPS45 |
| ENSG00000285427 | ENSG00000285441 | SOD2 |
| ENSG00000285427 | ENSG00000120437 | ACAT2 |
| ENSG00000285837 | ENSG00000285551 | AC067752.1 |
| ENSG00000285837 | ENSG00000138311 | ZNF365 |
| MSTRG.10663 | ENSG00000178974 | FBXO34 |
| MSTRG.1113 | ENSG00000269113 | TRABD2B |
| MSTRG.11455 | ENSG00000166166 | TRMT61A |
| MSTRG.11455 | ENSG00000075413 | MARK3 |
| MSTRG.11455 | ENSG00000166165 | CKB |
| MSTRG.12309 | ENSG00000047346 | FAM214A |
| MSTRG.1299 | ENSG00000162599 | NFIA |
| MSTRG.13915 | ENSG00000169203 | NPIPB12 |
| MSTRG.13915 | ENSG00000181625 | SLX1B |
| MSTRG.13915 | ENSG00000183336 | BOLA2 |
| MSTRG.13915 | ENSG00000261740 | BOLA2-SMG1P6 |
| MSTRG.13915 | ENSG00000213648 | SULT1A4 |
| MSTRG.14534 | ENSG00000140836 | ZFHX3 |
| MSTRG.15219 | ENSG00000141052 | MYOCD |
| MSTRG.15390 | ENSG00000083290 | ULK2 |
| MSTRG.15575 | ENSG00000176390 | CRLF3 |
| MSTRG.15636 | ENSG00000010244 | ZNF207 |
| MSTRG.15636 | ENSG00000108666 | C17orf75 |
| MSTRG.15963 | ENSG00000236383 | CCDC200 |
| MSTRG.16393 | ENSG00000062716 | VMP1 |
| MSTRG.16506 | ENSG00000261371 | PECAM1 |
| MSTRG.17503 | ENSG00000171791 | BCL2 |
| MSTRG.18183 | ENSG00000123146 | ADGRE5 |
| MSTRG.18516 | ENSG00000213096 | ZNF254 |
| MSTRG.19876 | ENSG00000158019 | BABAM2 |
| MSTRG.20290 | ENSG00000138069 | RAB1A |
| MSTRG.20295 | ENSG00000198369 | SPRED2 |
| MSTRG.20323 | ENSG00000115946 | PNO1 |
| MSTRG.20323 | ENSG00000221823 | PPP3R1 |
| MSTRG.20323 | ENSG00000273398 | AC017083.3 |
| MSTRG.20491 | ENSG00000176407 | KCMF1 |
| MSTRG.21281 | ENSG00000082438 | COBLL1 |
| MSTRG.21839 | ENSG00000178568 | ERBB4 |
| MSTRG.2200 | ENSG00000271383 | NBPF19 |
| MSTRG.2200 | ENSG00000286185 | AC242842.3 |
| MSTRG.2200 | ENSG00000286219 | AC242843.1 |
| MSTRG.2200 | ENSG00000263464 | PPIAL4C |
| MSTRG.22033 | ENSG00000153823 | PID1 |
| MSTRG.23270 | ENSG00000156273 | BACH1 |
| MSTRG.23270 | ENSG00000171189 | GRIK1 |
| MSTRG.23354 | ENSG00000159216 | RUNX1 |
| MSTRG.23459 | ENSG00000160193 | WDR4 |
| MSTRG.23459 | ENSG00000160194 | NDUFV3 |
| MSTRG.23968 | ENSG00000100154 | TTC28 |
| MSTRG.25357 | ENSG00000114541 | FRMD4B |
| MSTRG.25749 | ENSG00000160145 | KALRN |
| MSTRG.26375 | ENSG00000177565 | TBL1XR1 |
| MSTRG.26556 | ENSG00000145012 | LPP |
| MSTRG.27007 | ENSG00000169851 | PCDH7 |
| MSTRG.27075 | ENSG00000163694 | RBM47 |
| MSTRG.27087 | ENSG00000163697 | APBB2 |
| MSTRG.27946 | ENSG00000071205 | ARHGAP10 |
| MSTRG.28071 | ENSG00000145416 | MARCH1 |
| MSTRG.28521 | ENSG00000164190 | NIPBL |
| MSTRG.28651 | ENSG00000185305 | ARL15 |
| MSTRG.29420 | ENSG00000198108 | CHSY3 |
| MSTRG.29421 | ENSG00000198108 | CHSY3 |
| MSTRG.29858 | ENSG00000164574 | GALNT10 |
| MSTRG.29858 | ENSG00000037749 | MFAP3 |
| MSTRG.31086 | ENSG00000172348 | RCAN2 |
| MSTRG.31555 | ENSG00000009413 | REV3L |
| MSTRG.31799 | ENSG00000197442 | MAP3K5 |
| MSTRG.31802 | ENSG00000197442 | MAP3K5 |
| MSTRG.31846 | ENSG00000010818 | HIVEP2 |
| MSTRG.31970 | ENSG00000120254 | MTHFD1L |
| MSTRG.32018 | ENSG00000029639 | TFB1M |
| MSTRG.32018 | ENSG00000171217 | CLDN20 |
| MSTRG.32033 | ENSG00000175048 | ZDHHC14 |
| MSTRG.32574 | ENSG00000106355 | LSM5 |
| MSTRG.32574 | ENSG00000105778 | AVL9 |
| MSTRG.33836 | ENSG00000182158 | CREB3L2 |
| MSTRG.33865 | ENSG00000059377 | TBXAS1 |
| MSTRG.34642 | ENSG00000157110 | RBPMS |
| MSTRG.34763 | ENSG00000104365 | IKBKB |
| MSTRG.34846 | ENSG00000254087 | LYN |
| MSTRG.35307 | ENSG00000104517 | UBR5 |
| MSTRG.35883 | ENSG00000147862 | NFIB |
| MSTRG.36722 | ENSG00000148154 | UGCG |
| MSTRG.36868 | ENSG00000056586 | RC3H2 |
| MSTRG.37080 | ENSG00000130723 | PRRC2B |
| MSTRG.3823 | ENSG00000151474 | FRMD4A |
| MSTRG.4560 | ENSG00000156113 | KCNMA1 |
| MSTRG.4598 | ENSG00000188199 | NUTM2B |
| MSTRG.4718 | ENSG00000180628 | PCGF5 |
| MSTRG.5019 | ENSG00000151532 | VTI1A |
| MSTRG.5029 | ENSG00000148737 | TCF7L2 |
| MSTRG.6012 | ENSG00000198561 | CTNND1 |
| MSTRG.6012 | ENSG00000254732 | AP001931.1 |
| MSTRG.6012 | ENSG00000254462 | TMX2-CTNND1 |
| MSTRG.628 | ENSG00000174950 | CD164L2 |
| MSTRG.628 | ENSG00000181773 | GPR3 |
| MSTRG.628 | ENSG00000158195 | WASF2 |
| MSTRG.6846 | ENSG00000184384 | MAML2 |
| MSTRG.6846 | ENSG00000087053 | MTMR2 |
| MSTRG.7089 | ENSG00000168092 | PAFAH1B2 |
| MSTRG.7404 | ENSG00000151067 | CACNA1C |
| MSTRG.8487 | ENSG00000196935 | SRGAP1 |
| MSTRG.8904 | ENSG00000171310 | CHST11 |
| MSTRG.9103 | ENSG00000135090 | TAOK3 |
| MSTRG.9634 | ENSG00000102804 | TSC22D1 |
| ENSG00000261215 | ENSG00000137070 | IL11RA |
| ENSG00000247572 | ENSG00000131732 | ZCCHC9 |
| ENSG00000196295 | ENSG00000106105 | GARS |
| ENSG00000259583 | ENSG00000154237 | LRRK1 |
| ENSG00000272994 | ENSG00000135974 | C2orf49 |
| ENSG00000245573 | ENSG00000148943 | LIN7C |
| ENSG00000236859 | ENSG00000074054 | CLASP1 |
| ENSG00000228889 | ENSG00000134882 | UBAC2 |
| ENSG00000285967 | ENSG00000164190 | NIPBL |
| ENSG00000251652 | ENSG00000178222 | RNF212 |
| ENSG00000279943 | ENSG00000145476 | CYP4V2 |
| ENSG00000272716 | ENSG00000152683 | SLC30A6 |
| ENSG00000272092 | ENSG00000147548 | NSD3 |
| ENSG00000227540 | ENSG00000182180 | MRPS16 |
| ENSG00000183250 | ENSG00000160256 | FAM207A |
| ENSG00000235257 | ENSG00000144677 | CTDSPL |
| ENSG00000274925 | ENSG00000155592 | ZKSCAN2 |
| ENSG00000242082 | ENSG00000128253 | RFPL2 |
| ENSG00000279207 | ENSG00000264364 | DYNLL2 |
| ENSG00000271576 | ENSG00000142875 | PRKACB |
| ENSG00000248932 | ENSG00000184432 | COPB2 |
| ENSG00000260630 | ENSG00000167508 | MVD |
| ENSG00000263843 | ENSG00000125457 | MIF4GD |
| ENSG00000279539 | ENSG00000105723 | GSK3A |
| ENSG00000279742 | ENSG00000150676 | CCDC83 |
| ENSG00000250241 | ENSG00000138650 | PCDH10 |
| ENSG00000228956 | ENSG00000131374 | TBC1D5 |
| ENSG00000235358 | ENSG00000010803 | SCMH1 |
| MSTRG.6130 | ENSG00000176485 | PLA2G16 |
| ENSG00000269900 | ENSG00000159884 | CCDC107 |
| ENSG00000212694 | ENSG00000139718 | SETD1B |
| ENSG00000239665 | ENSG00000165630 | PRPF18 |
| ENSG00000267296 | ENSG00000245848 | CEBPA |
| ENSG00000267100 | ENSG00000129351 | ILF3 |
| ENSG00000248101 | ENSG00000105270 | CLIP3 |
| ENSG00000275897 | ENSG00000136444 | RSAD1 |
| ENSG00000261971 | ENSG00000162069 | BICDL2 |
| ENSG00000279035 | ENSG00000125449 | ARMC7 |
| ENSG00000231437 | ENSG00000171385 | KCND3 |
| ENSG00000270361 | ENSG00000163344 | PMVK |
| ENSG00000232533 | ENSG00000159840 | ZYX |
| ENSG00000183250 | ENSG00000160255 | ITGB2 |
| ENSG00000269900 | ENSG00000137135 | ARHGEF39 |
| ENSG00000280277 | ENSG00000111880 | RNGTT |
| ENSG00000261357 | ENSG00000167186 | COQ7 |
| ENSG00000268858 | ENSG00000101150 | TPD52L2 |
| ENSG00000228242 | ENSG00000170876 | TMEM43 |
| ENSG00000275897 | ENSG00000167107 | ACSF2 |
| ENSG00000279569 | ENSG00000157368 | IL34 |
