# Supplementary material for: Transcriptomic and Proteomic Profiling of Human Stable and Unstable Carotid Atherosclerotic Plaques
Source: Front Genet. 2021 Nov 4;12:755507. doi: 10.3389/fgene.2021.755507 (PMC8599967; doi:10.3389/fgene.2021.755507)
Supplement: Supplementary file 6 [file Table4.docx]

Table 4 circRNA originated genes

| **circRNA** | **SOURCE GENE ID** | **Name** |
| --- | --- | --- |
| hsacirc_018650 | ENSG00000007341 | ST7L |
| hsacirc_002651 | ENSG00000031003 | FAM13B |
| hsacirc_007292 | ENSG00000068305 | MEF2A |
| hsacirc_012286 | ENSG00000073417 | PDE8A |
| hsacirc_002622 | ENSG00000073921 | PICALM |
| hsacirc_002439 | ENSG00000074054 | CLASP1 |
| hsacirc_012506 | ENSG00000075151 | EIF4G3 |
| hsacirc_030053 | ENSG00000077943 | ITGA8 |
| hsacirc_013094 | ENSG00000082701 | GSK3B |
| hsacirc_033511 | ENSG00000082898 | XPO1 |
| hsacirc_004256 | ENSG00000083093 | PALB2 |
| hsacirc_018644 | ENSG00000085365 | SCAMP1 |
| hsacirc_004372 | ENSG00000095139 | ARCN1 |
| hsacirc_000411 | ENSG00000097033 | SH3GLB1 |
| hsacirc_000128 | ENSG00000100099 | HPS4 |
| hsacirc_025501 | ENSG00000100403 | ZC3H7B |
| hsacirc_029512 | ENSG00000100485 | SOS2 |
| hsacirc_054743 | ENSG00000100629 | CEP128 |
| hsacirc_041066 | ENSG00000101040 | ZMYND8 |
| hsacirc_010308 | ENSG00000101639 | CEP192 |
| hsacirc_014201 | ENSG00000101871 | MID1 |
| hsacirc_036835 | ENSG00000102780 | DGKH |
| hsacirc_037511 | ENSG00000102781 | KATNAL1 |
| hsacirc_023865 | ENSG00000103150 | MLYCD |
| hsacirc_040556 | ENSG00000105323 | HNRNPUL1 |
| hsacirc_052168 | ENSG00000106853 | PTGR1 |
| hsacirc_021753 | ENSG00000109323 | MANBA |
| hsacirc_012229 | ENSG00000111252 | SH2B3 |
| hsacirc_013041 | ENSG00000114861 | FOXP1 |
| hsacirc_014488 | ENSG00000115183 | TANC1 |
| hsacirc_020492 | ENSG00000115677 | HDLBP |
| hsacirc_004306 | ENSG00000115947 | ORC4 |
| hsacirc_042370 | ENSG00000118454 | ANKRD13C |
| hsacirc_004618 | ENSG00000120071 | KANSL1 |
| hsacirc_027196 | ENSG00000123066 | MED13L |
| hsacirc_019089 | ENSG00000123094 | RASSF8 |
| hsacirc_014814 | ENSG00000123104 | ITPR2 |
| hsacirc_053072 | ENSG00000124532 | MRS2 |
| hsacirc_043717 | ENSG00000131018 | SYNE1 |
| hsacirc_035602 | ENSG00000131196 | NFATC1 |
| hsacirc_045048 | ENSG00000131626 | PPFIA1 |
| hsacirc_035969 | ENSG00000131724 | IL13RA1 |
| hsacirc_011656 | ENSG00000132676 | DAP3 |
| hsacirc_036080 | ENSG00000133026 | MYH10 |
| hsacirc_042514 | ENSG00000133302 | SLF1 |
| hsacirc_021360 | ENSG00000134313 | KIDINS220 |
| hsacirc_037442 | ENSG00000134909 | ARHGAP32 |
| hsacirc_045073 | ENSG00000134954 | ETS1 |
| hsacirc_022504 | ENSG00000135749 | PCNX2 |
| hsacirc_021900 | ENSG00000136146 | MED4 |
| hsacirc_019756 | ENSG00000136478 | TEX2 |
| hsacirc_002641 | ENSG00000138380 | CARF |
| hsacirc_010738 | ENSG00000138735 | PDE5A |
| hsacirc_010412 | ENSG00000139725 | RHOF |
| hsacirc_043499 | ENSG00000142347 | MYO1F |
| hsacirc_025902 | ENSG00000144815 | NXPE3 |
| hsacirc_047450 | ENSG00000144824 | PHLDB2 |
| hsacirc_017282 | ENSG00000148634 | HERC4 |
| hsacirc_005332 | ENSG00000148925 | BTBD10 |
| hsacirc_008209 | ENSG00000149311 | ATM |
| hsacirc_034555 | ENSG00000150630 | VEGFC |
| hsacirc_019932 | ENSG00000150938 | CRIM1 |
| hsacirc_013530 | ENSG00000151552 | QDPR |
| hsacirc_013598 | ENSG00000151575 | TEX9 |
| hsacirc_040211 | ENSG00000156304 | SCAF4 |
| hsacirc_037940 | ENSG00000156931 | VPS8 |
| hsacirc_001638 | ENSG00000157184 | CPT2 |
| hsacirc_007214 | ENSG00000159128 | IFNGR2 |
| hsacirc_021099 | ENSG00000162607 | USP1 |
| hsacirc_042677 | ENSG00000162929 | KIAA1841 |
| hsacirc_014106 | ENSG00000163162 | RNF149 |
| hsacirc_051322 | ENSG00000164953 | TMEM67 |
| hsacirc_028523 | ENSG00000166435 | XRRA1 |
| hsacirc_049639 | ENSG00000169410 | PTPN9 |
| hsacirc_054182 | ENSG00000170037 | CNTROB |
| hsacirc_012069 | ENSG00000178385 | PLEKHM3 |
| hsacirc_014037 | ENSG00000180357 | ZNF609 |
| hsacirc_026080 | ENSG00000182022 | CHST15 |
| hsacirc_020435 | ENSG00000182158 | CREB3L2 |
| hsacirc_017172 | ENSG00000196782 | MAML3 |
| hsacirc_027103 | ENSG00000204130 | RUFY2 |
| hsacirc_011188 | ENSG00000204186 | ZDBF2 |
| hsacirc_012765 | ENSG00000214595 | EML6 |
| hsacirc_035172 | ENSG00000228144 | AC078927.1 |
| hsacirc_005476 | ENSG00000230006 | - |
| hsacirc_011690 | ENSG00000244754 | N4BP2L2 |
| hsacirc_016301 | ENSG00000254087 | LYN |
| hsacirc_010153 | ENSG00000268015 | - |
| hsacirc_052530 | ENSG00000285053 | TBCE |
| hsacirc_013041 | ENSG00000285708 | AC097634.4 |
| hsacirc_035172 | ENSG00000155957 | TMBIM4 |
| hsacirc_017282 | ENSG00000226318 | - |
| hsacirc_049639 | ENSG00000259931 | - |
| hsacirc_023865 | ENSG00000260300 | AC009119.2 |
| hsacirc_052530 | ENSG00000284770 | TBCE |
| hsacirc_035969 | ENSG00000250995 | - |
