# Supplementary material for: Transcriptomic and Proteomic Profiling of Human Stable and Unstable Carotid Atherosclerotic Plaques
Source: Front Genet. 2021 Nov 4;12:755507. doi: 10.3389/fgene.2021.755507 (PMC8599967; doi:10.3389/fgene.2021.755507)
Supplement: Supplementary file 7 [file Table6.docx]

**Table 6 The list of clustered proteins identified by MCODE**

|  | **Gene symbol** | **MCODE score** | **Biological functions of these genes** |
| --- | --- | --- | --- |
| **Cluster 1** | TPM4  TPM1  SORBS1  MYL6  HEL-S-273  CALD1  ACTG2  tmp_locus_29 | 4.5 | The contractile function of smooth muscles and the cytoskeleton of non-muscle cells;  The signaling and stimulation of insulin; |
| **Cluster 2** | SAA4  PON1  HPR  APOM  APOL1  APOB | 3.57 | Lactonase and ester hydrolase activity; Metabolism and transportation of lipoproteins; |
| **Cluster 3** | HLA-DRB1  HLA-DPB1  HLA-DPA1  HLA-C  HLA-B  HLA-A | 2.67 | Play a central role in the immune system by presenting peptides derived from extracellular proteins |
| **Cluster 4** | U2AF2  TRA2B  SRSF6  SRSF1 | 1.8 | The mRNA processing, splicing patterns, and gene expression. |
